# An ecological study of socioeconomic predictors in detection of COVID-19 cases across neighborhoods in New York City

**DOI:** 10.1186/s12916-020-01731-6

**Published:** 2020-09-04

**Authors:** Richard S. Whittle, Ana Diaz-Artiles

**Affiliations:** 1 Department of Aerospace Engineering, Texas A&M University, College Station, TX USA; 2 International Space University, Illkirch-Graffenstaden, France

**Keywords:** COVID-19, Positivity rate, Socioeconomic factors, Besag-York-Mollié model, Youth dependency, Population density, Race, Income

## Abstract

**Background:**

New York City was the first major urban center of the COVID-19 pandemic in the USA. Cases are clustered in the city, with certain neighborhoods experiencing more cases than others. We investigate whether potential socioeconomic factors can explain between-neighborhood variation in the COVID-19 test positivity rate.

**Methods:**

Data were collected from 177 Zip Code Tabulation Areas (ZCTA) in New York City (99.9% of the population). We fit multiple Bayesian Besag-York-Mollié (BYM) mixed models using positive COVID-19 tests as the outcome, a set of 11 representative demographic, economic, and health-care associated ZCTA-level parameters as potential predictors, and the total number of COVID-19 tests as the exposure. The BYM model includes both spatial and nonspatial random effects to account for clustering and overdispersion.

**Results:**

Multiple regression approaches indicated a consistent, statistically significant association between detected COVID-19 cases and dependent children (under 18 years old), population density, median household income, and race. In the final model, we found that an increase of only 5% in young population is associated with a 2.3% increase in COVID-19 positivity rate (95% confidence interval (CI) 0.4 to 4.2%, *p*=0.021). An increase of 10,000 people per km^2^ is associated with a 2.4% (95% CI 0.6 to 4.2%, *p*=0.011) increase in positivity rate. A decrease of $10,000 median household income is associated with a 1.6% (95% CI 0.7 to 2.4%, *p*<0.001) increase in COVID-19 positivity rate. With respect to race, a decrease of 10% in White population is associated with a 1.8% (95% CI 0.8 to 2.8%, *p*<0.001) increase in positivity rate, while an increase of 10% in Black population is associated with a 1.1% (95% CI 0.3 to 1.8%, *p*<0.001) increase in positivity rate. The percentage of Hispanic (*p*=0.718), Asian (*p*=0.966), or Other (*p*=0.588) populations were not statistically significant factors.

**Conclusions:**

Our findings indicate associations between neighborhoods with a large dependent youth population, densely populated, low-income, and predominantly black neighborhoods and COVID-19 test positivity rate. The study highlights the importance of public health management during and after the current COVID-19 pandemic. Further work is warranted to fully understand the mechanisms by which these factors may have affected the positivity rate, either in terms of the true number of cases or access to testing.

## Background

On 21 January 2020, the first case of coronavirus disease 2019 (COVID-19) in the USA was reported in Washington State [1]. The first case was not reported in New York state until 1 March 2020 [2]. By the time the World Health Organization (WHO) declared a global pandemic on 11 March 2020, there were 345 cases in New York City (NYC), and this number skyrocketed to nearly 18,000 cases just 2 weeks later [2, 3]. NYC rapidly became the epicenter of the pandemic in the USA, with a transmission rate five times higher than the rest of the country, and over a third of all confirmed national cases by early April [4].

During a pandemic, there is likely to be large variation in both disease transmission and disease testing between regions [5]. These two factors cause large variation in disease reporting between different areas [6]. This is particularly true in the early stages of the outbreak, before disease testing has become widespread and standardized.

Contemporary and historical studies on previous pandemics, including H1N1 pandemics in 1918 and 2009, suggest that socioeconomic factors on a national level can affect detection rates and medical outcomes [7–9]. Thus, socioeconomic factors such as young or old populations, race, affluence, inequality, poverty, unemployment, insurance, or access to healthcare may account for differences in reported cases of COVID-19 between neighborhoods in NYC.

The aim of this ecological study was to identify potential neighbourhood-level socioeconomic determinants of the COVID-19 test positivity rate and explain between-neighborhood variation during the early, exponential growth stage of the pandemic in NYC: from the first detected case in 1 March until 5 April 2020.

## Methods

### Data collection

Data on positive COVID-19 cases were collected from NYC Department of Health and Mental Hygiene (DOHMH) Incident Command System for COVID-19 Response (Surveillance and Epidemiology Branch in collaboration with Public Information Office Branch) [2]. Since the NYC DOHMH was discouraging people with mild to moderate symptoms from being tested during the time period covered, the data primarily represents people with more severe illness. Since at the time of writing the pandemic is still ongoing, data were taken at a snapshot on 5 April2020. This date was chosen to cover the first month of the pandemic in NYC, since understanding early etiology of the pandemic and local influences is important in helping to inform future management [10]. Data were a cumulative count up to and including 5 April 2020. On this date, NYC had a cumulative total of 64,955 cases [11], including deaths and hospitalizations.

**Fig. 1:**
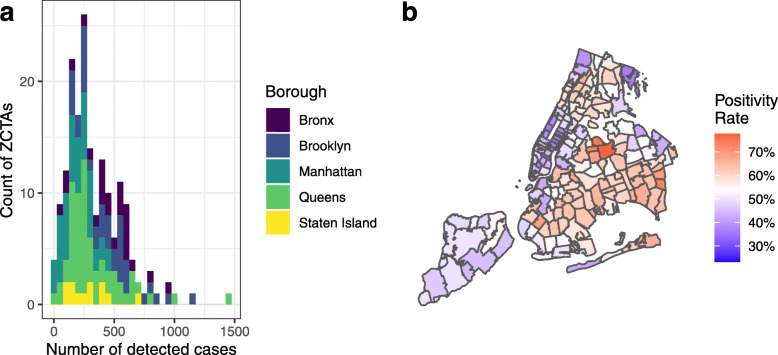
New York City detected COVID-19 cases by Zip Code Tabulation Area (ZCTA). As at 5 April 2020. **a** Histogram of detected cases by ZCTA, grouped by borough. **b** Positivity rate, or detected cases as a percentage of total tests

The available dataset included 64,512 cases (99.3% of total cases), with each case representing a positive diagnosis of COVID-19 along with the patient’s Zip Code Tabulation Area (ZCTA). ZCTAs are generalized areal representations of United States Postal Service (USPS) Zip Code service areas. ZCTAs were the areas in which patients reported their home address, as opposed to either where they became symptomatic or where they reported for testing/treatment. The area of interest covered 177 ZCTAs within NYC, from 10001 (Chelsea, Manhattan) to 11697 (Breezy Point, Queens). Of these cases, there were 4712 where the patient ZCTA was unknown and thus these cases were discarded, leaving 59,800 cases (92.1% of total cases). Note that this total is not meant to be an indicator of the total number of COVID-19 cases at this time, rather the count of *detected* cases. The dataset also included the total number of tests conducted by ZCTA. Figure 1a shows a histogram of detected cases by ZCTA as at 5 April 2020, grouped by the five boroughs of NYC (Bronx, Brooklyn, Manhattan, Queens, and Staten Island); Fig. 1b displays these cases on a map as a percentage of total COVID-19 tests performed.

Data on potential predictor variables were collected from the United States Census Bureau American Community Survey (ACS). ACS is a continuous sample survey of 3.5 million households every year including questions beyond the decadal census on subjects such as education, employment, internet access, and transportation. Data were collected at ZCTA level from the ACS 2014-2018 5-year estimate [12], which is the most recent publicly available.

The 5-year estimate was chosen instead of the most recent 1-year estimate because the latter was not available in an aggregated form at ZCTA level and only at the Public Use Microdata Area (PUMA) level. PUMAs contain multiple ZCTAs, but for the most part, the boundaries are not equivalent to the ZCTA boundaries used in the COVID-19 dataset. In addition, while the 5-year estimate is less current, it has a smaller margin of error than the 1-year estimate and greater statistical reliability for small geographic areas. To further understand any potential differences, we compared a sample of the ACS 5-year estimate with the most recent available 1-year estimate in an area where these two area systems overlap: Rockaway Peninsula, where PUMA area 3604114 (*NYC Queens Community District 14: Far Rockaway, Breezy Point & Broad Channel PUMA*) overlaps with ZCTAs 11691, 11692, 11693, 11694, and 11697. We found agreement in all parameters included in our study within the margins of error of the survey.

### Demographic parameters

**Fig. 2:**
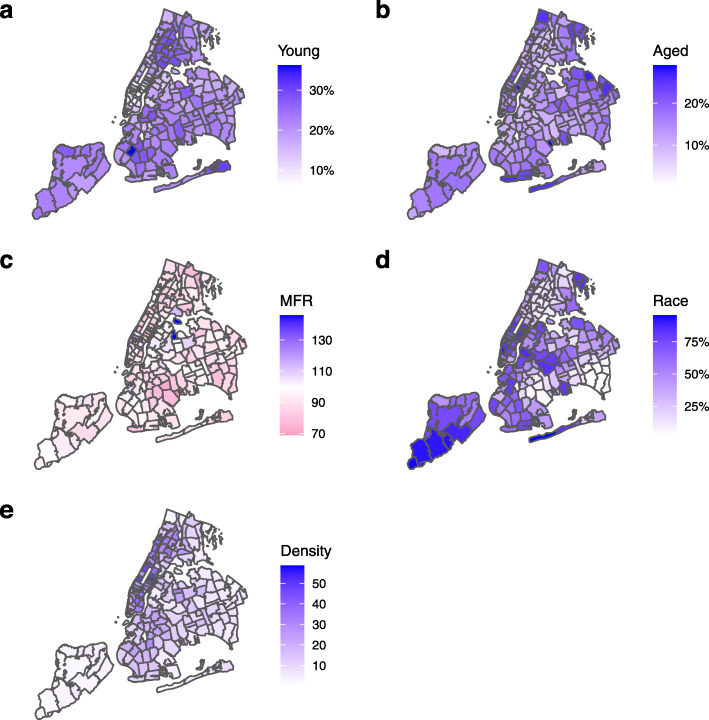
New York City demographic predictors by Zip Code Tabulation Area (ZCTA). Data based on American Community Survey (ACS) 2018 5-year estimates. **a***Young*, percentage of population aged under 18. **b***Aged*, percentage of population aged 65+. **c***MFR*, males per 100 females. **d***Race*, percentage of population that identify as white (alone or in combination with another race). **e***Density*, population density in ’000s persons per km^2^

Five demographic parameters were included in the study: percentage of young dependent population, *Young*; percentage of aged population, *Aged*; males per 100 females, *MFR*; percentage of the population identifying as white, *Race*; and population density, *Density*. Young dependent population was defined as the percentage of the total population aged under 18. Aged population was the percentage of the total population 65+. These are both typically economically inactive populations. The increased severity of COVID-19 with increasing age has been well documented [13], and there has been recent evidence of asymptomatic carrier transmission particularly among young people [14, 15]. Males per 100 females was chosen to capture the balance of sex in the population. We were interested in whether sex differences lead to significant variation in detected cases. Some reports suggest a racial disparity in case detection rates across the USA. A report from NYU Furman Center for housing, neighborhoods, and urban policy suggests mortality rates are higher among the city’s “Hispanic, Black, and non-Hispanic/Latino: Other” populations [16]. For the present study, we initially chose to include the percentage of the population that identify as white (alone or in combination with another race) as a combined indicator of all minority populations. Thus, we united multiple races with distinct levels of COVID-19 incidence [17] into a single metric for model building purposes (i.e., white vs non-white). Then, we also considered a more detailed analysis of the racial structure of neighborhoods by further analyzing five separate racial groups: White, Black, Hispanic, Asian, and Other (including American Indian and Alaska Native, Native Hawaiian and Other Pacific Islanders, Caribbean, and Mixed Race). Finally, we also included population density based on studies of the 2008 H1N1 Influenza pandemic highlighting population density as a significant risk factor for transmission [18]. The distributions of demographic predictors in the area of interest are shown in Fig. 2.

### Economic parameters

**Fig. 3:**
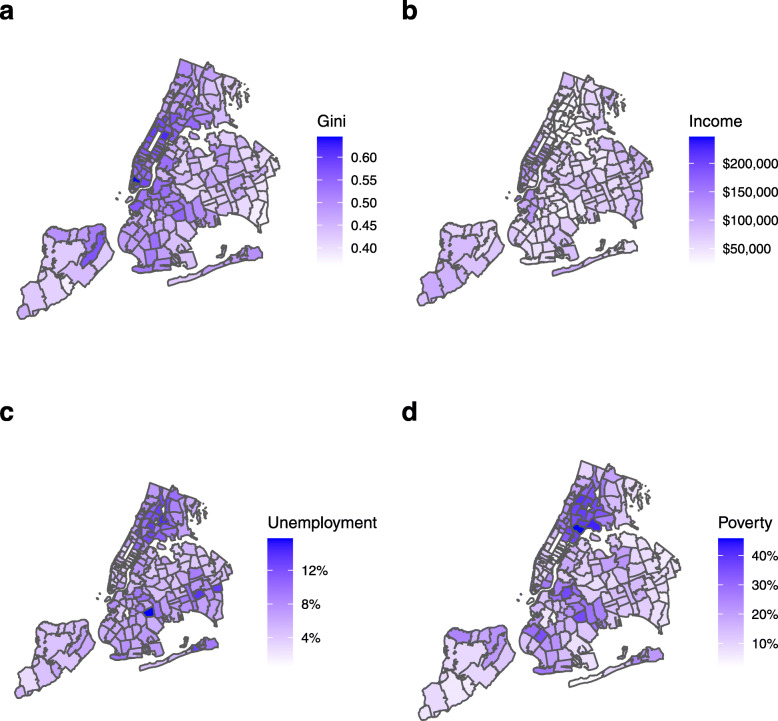
New York City economic predictors by Zip Code Tabulation Area (ZCTA). Data based on American Community Survey (ACS) 2018 5-year estimates. **a***Gini*, Gini index. **b***Income*, median household income. **c***Unemployment*, percentage of working age population unemployed. **d***Poverty*, percentage of total population living below the poverty threshold

Four economic parameters were included in the study: Gini index, *Gini*; median household income, *Income*; percentage of labor force unemployed, *Unemployment*; and percentage of population living below the poverty threshold, *Poverty*. Gini index is a measure of economic inequality ranging from 0 to 1. An index of 0 indicates all the wealth in an area is divided equally among the population, while an index of 1 indicates all the wealth is held by one individual. While some studies have argued against the adverse effects of unequal income [19], an association has been demonstrated between inequality and population health [20]. We also included household income, which was a significant predictor for hospitalizations in the 2009 influenza pandemic [21]. Specifically, in the present study, we use median household income as a ZCTA-level predictor. Finally, unemployment and poverty both have documented association with health outcomes, including in pandemic scenarios [22, 23]. While there is some level of collinearity between these two variables, we include both as one relates to the economically active labor force whereas the other relates to the total population. The distributions of economic predictors in the area of interest are shown in Fig. 3.

### Health parameters

**Fig. 4:**
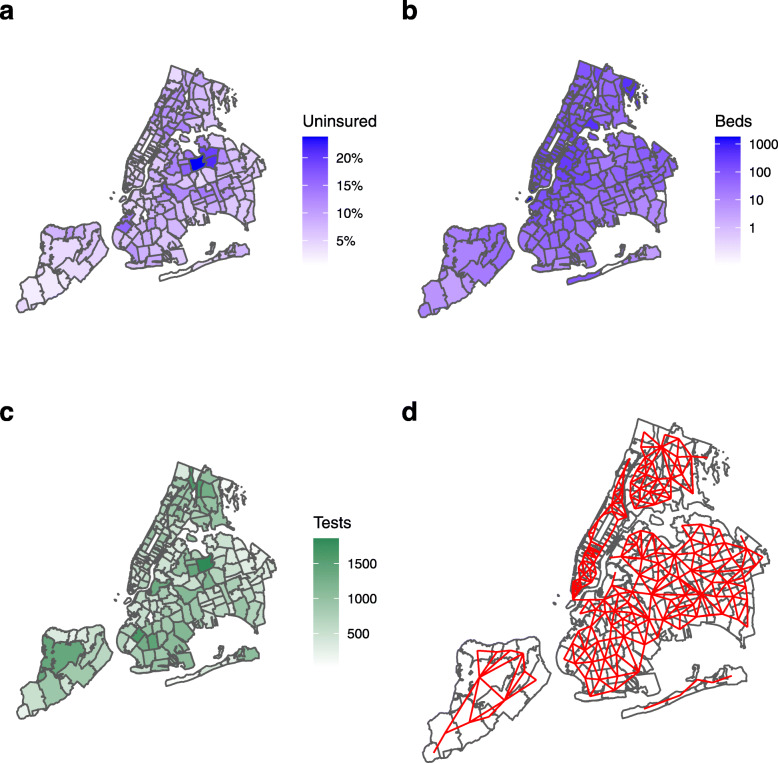
New York City health predictors by Zip Code Tabulation Area (ZCTA). Data based on American Community Survey (ACS) 2018 5-year estimates. **a***Uninsured*, percentage of total population uninsured. **b***Beds*, total number of hospital beds per 1000 people within 5 km. **c** Total COVID-19 tests (exposure). **d** neighborhood connectivity

Two parameters related to healthcare access were included in the study: percentage of population uninsured, *Uninsured*; and total number of hospital bed per 1000 people within 5 km, *Beds*. It has been documented that lack of insurance can delay access to timely healthcare, particularly during pandemics [24]. We hypothesized that this parameter could affect virus transmission and/or access to testing, therefore affecting detection rates. Finally, we chose *Beds* as a parameter related to proximity to healthcare, which has been shown to be inversely associated with adverse outcomes in other geospatial public health studies [25]. For a city containing multiple hospitals such as NYC, we defined a proximity metric in this study as population normalized number of hospital beds within 5 km. This predictor was chosen as a secondary metric reflecting general societal access to healthcare and localized investment in healthcare infrastructure. The distributions of health related predictors in the area of interest are shown in Fig. 4a, b. Figure 4 also shows two other factors used in the model; Fig. 4c shows the number of tests conducted in each ZCTA used as the model exposure, and Fig. 4d shows the neighborhood connectivity between ZCTAs, used for spatial effects.

### Statistical analysis

#### Base model

Prior to analysis of potential predictors, we considered multiple base regression models. Given the significant spatial correlation in the present case data as evidenced by the Moran Index, *I*(176)=0.642, *p*<0.0005 [26], we explored potential regression models both with and without spatial effects. We compared four base models (no predictors): (1) a Poisson model with random intercept, (2) a Poisson Besag-York-Mollié (BYM) model [27], (3) a negative binomial model with random intercept, and (4) a negative binomial BYM model. The BYM model is the union of a Besag model [28], *υ*, and a nonspatial random effect, *ν*, such that the linear predictor for spatial unit *i*, *η*_*i*_, is given by Eq 1:

1 $$\begin{array}{@{}rcl@{}}  \eta_{i}=\upsilon_{i}+\nu_{i} \end{array} $$

where *υ*_*i*_ has an intrinsic conditional autoregressive (ICAR) structure [29]. We used the reparameterization of the BYM model proposed by Riebler et al. [30], known as the BYM2 model and shown in Eq 2:

2 $$\begin{array}{@{}rcl@{}} \upsilon_{i}+\nu_{i}=\frac{1}{\sqrt{\tau_{\gamma}}}\left({\sqrt{\varphi}\upsilon_{i}^{*}}+\sqrt{1-\varphi}\nu_{i}^{*}\right)  \end{array} $$

where *τ*_*γ*_ is the overall precision hyperparameter, *φ*∈[0,1] is the mixing hyperparameter representing the proportional division of variance between the spatial and nonspatial effects, *υ*^∗^ is the spatial (ICAR) effect with a scaling factor such that Var(*υ*^∗^)≈1, and *ν*^∗^ is the nonspatial random-effect with *ν*^∗^∼N(0,1). Penalized complexity (PC) priors are applied to hyperparameters *τ*_*γ*_ and *φ* (compared to log-gamma priors in the random intercept model) [31]. All four models used ZCTA total number of COVID-19 tests as the exposure and a log-link function. We selected the model with the lowest deviance information criterion (DIC) [32], representing the best trade-off between model fit and complexity.

Characteristics for the four base models examined, including hyperparameters, are shown in Table 1. The two Poisson models (models 1 and 2) had significantly lower DIC than the negative binomial models. The Poisson BYM2 model (model 2) was marginally better than the simple random effect model (model 1). Thus, the Poisson BYM2 model was selected and used for all future analyses and regressions.

**Table 1:** Characteristics of four different base models (no predictors). Lower deviance information criterion (DIC) represents a better trade off between model fit and complexity. Models 1 and 3 have a random intercept; models 2 and 4 follow a BYM2 structure. $D\left (\overline \theta \right)$, deviance of mean model parameters *θ*; *p*_*D*_, effective number of parameters

Model	Distribution	Parameters	Hyperparameters	$D\left (\overline \theta \right)$	*p*_*D*_	DIC
Model 1^*^	Poisson	*β*_0_, *ν*_*i*_	*τ*_*ν*_	1346.53	149.6	1645.73
Model 2^**^	Poisson	*β*_0_, $\upsilon _{i}^{*}$, $\nu _{i}^{*}$	*τ*_*γ*_, *φ*	1362.37	124.68	1611.73
Model 3^†^	Negative binomial	*β*_0_, *ν*_*i*_	*n*, *τ*_*ν*_	1855.47	3.30	1862.07
Model 4^‡^	Negative binomial	*β*_0_, $\upsilon _{i}^{*}$, $\nu _{i}^{*}$	*n*, *τ*_*γ*_, *φ*	1455.71	103.58	1662.87

^*^Model 1: *y*_*i*_|*λ*_*i*_∼Pois(*λ*_*i*_), log(*λ*_*i*_)=*η*_*i*_+log(*E*_*i*_)=*β*_0_+*ν*_*i*_+log(*E*_*i*_)

^**^Model 2: *y*_*i*_|*λ*_*i*_∼Pois(*λ*_*i*_), $\log \left (\lambda _{i}\right)=\eta _{i}+\log \left (E_{i}\right)=\beta _{0}+\frac {1}{\sqrt {\tau _{\gamma }}}\left ({\sqrt {\varphi }\upsilon _{i}^{*}}+\sqrt {1-\varphi }\nu _{i}^{*}\right)+\log \left (E_{i}\right)$

^†^Model 3: *y*_*i*_|*λ*_*i*_∼NegBin(*n*,*λ*_*i*_), log(*λ*_*i*_)=*η*_*i*_+log(*E*_*i*_)=*β*_0_+*ν*_*i*_+log(*E*_*i*_)

^‡^Model 4: *y*_*i*_|*λ*_*i*_∼NegBin(*n*,*λ*_*i*_), $\log \left (\lambda _{i}\right)=\eta _{i}+\log \left (E_{i}\right)=\beta _{0}+\frac {1}{\sqrt {\tau _{\gamma }}}\left ({\sqrt {\varphi }\upsilon _{i}^{*}}+\sqrt {1-\varphi }\nu _{i}^{*}\right)+\log \left (E_{i}\right)$

Symbols: *y*_*i*_, count of cases in Zip Code Tabulation Area (ZCTA) *i*; *λ*_*i*_, expected cases in ZCTA *i*; *E*_*i*_, number of total COVID-19 tests in ZCTA *i*; *η*_*i*_, linear predictor for ZCTA *i*; *β*_0_, intercept; *ν*_*i*_, nonspatial random-effect; $\nu _{i}^{*}$, scaled nonspatial random-effect; $\upsilon _{i}^{*}$, scaled spatial random-effect with intrinsic conditional autoregressive structure; *τ*_*ν*_, precision for nonspatial random effect, log-gamma prior; *τ*_*γ*_, overall precision, penalized complexity (PC) prior; *φ*, mixing parameter, PC prior; *n*, overdispersion parameter, PC gamma prior

#### Adding predictors

Multiple regression models were built using a method adjusted from Nikolopoulos et al. [33]. In the univariable models, we considered each predictor variable separately (i.e., one model per variable). In the multivariable model, we considered all predictor variables together. We further built a partial multivariable model using only those predictors that were significant in the univariable models. Finally, we built a model using stepwise backwards elimination procedure, starting with the fully saturated model and removing the least significant predictor until we were left with a model containing only significant predictors [33]. In all cases, the expected number of detected COVID-19 cases in ZCTA *i*, *λ*_*i*_, was represented by Eq 3:

3 $$\begin{array}{*{20}l} \log\left(\lambda_{i}\right)=&\eta_{i}+\log\left(E_{i}\right)=\beta_{0}+\sum_{p=1}^{P}{\beta_{p} x_{ip}} \\ &+\frac{1}{\sqrt{\tau_{\gamma}}}\left({\sqrt{\varphi}\upsilon_{i}^{*}}+\sqrt{1-\varphi}\nu_{i}^{*}\right)+\log\left(E_{i}\right)  \end{array} $$

where *E*_*i*_ is the exposure (i.e., number of tests) for ZCTA *i*, *β*_0_ is the intercept, *β*_*p*_ is coefficient of the fixed effect for predictor *p*∈{1...*P*}, *x*_*ip*_ is the value of predictor *p* in ZCTA *i*, and the spatial and nonspatial random effects for ZCTA *i* are described by the BYM2 model detailed above. Vague Gaussian priors are assumed on all *β*.

#### Model fitting

Regression estimates are presented as mean and 95% confidence intervals (CI) sampled from the posterior marginal distribution, along with corresponding *p* values. We used posterior tail-area of the fixed effects as a Bayesian counterpart to *p* value [34]. All significance levels were two-sided with *p* value of <0.05 considered statistically significant. Statistical analysis was performed using R Statistical Software (version 4.0.0; R Foundation for Statistical Computing, Vienna, Austria). Models were fit via integrated nested Laplace approximation [35] using the R-INLA package [36]. Vague priors were assumed on all models.

## Results

As at 5 April 2020, 59,800 COVID-19 cases were reported with a known ZCTA. The highest number of cases in any particular ZCTA was 1,446 in ZCTA 11368 (Corona, Queens), while the lowest was 7 in ZCTA 10006 (Wall St, Manhattan). With respect to the proportion of tests returned positive, these two ZCTAs also had the highest and lowest positivity rates (23.33% and 77.70% respectively). On average, 0.71% of the total NYC population had tested positive for COVID-19, with 56.47% of total tests conducted returning a positive result.

### Base model

**Fig. 5:**
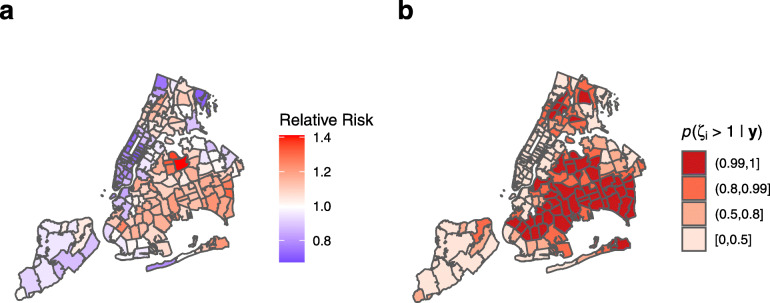
Disease mapping model for COVID-19 cases in New York City by Zip Code Tabulation Area (ZCTA). As at April 5, 2020, using base Poisson BYM2 model with no predictors. The area specific relative risk is multiplied by the total population average COVID-19 positivity rate (56.47%) to give the area specific positivity rate. **a** Area-specific relative risk, *ζ*_*i*_. **b** Posterior probability for relative risk, *p*(*ζ*_*i*_>1|**y**)

Using the base model, Fig. 5a shows the area specific relative risk *ζ*_*i*_. A value of *ζ*_*i*_=1 represents a positivity rate in line with the total population average (56.47% of total COVID-19 tests in area *i* have returned positive), while, for example, a value of *ζ*_*i*_=1.2 represents a positivity rate 1.2 times the total population average (67.76%). Figure 5b shows the posterior probability that the relative risk is greater than 1, *p*(*ζ*_*i*_>1|**y**). The map shows that the highest risk area is Corona, Queens, with three other significant clusters in the Bronx, Southeast Queens, and Southwest Brooklyn.

### Adding predictors

**Fig. 6:**
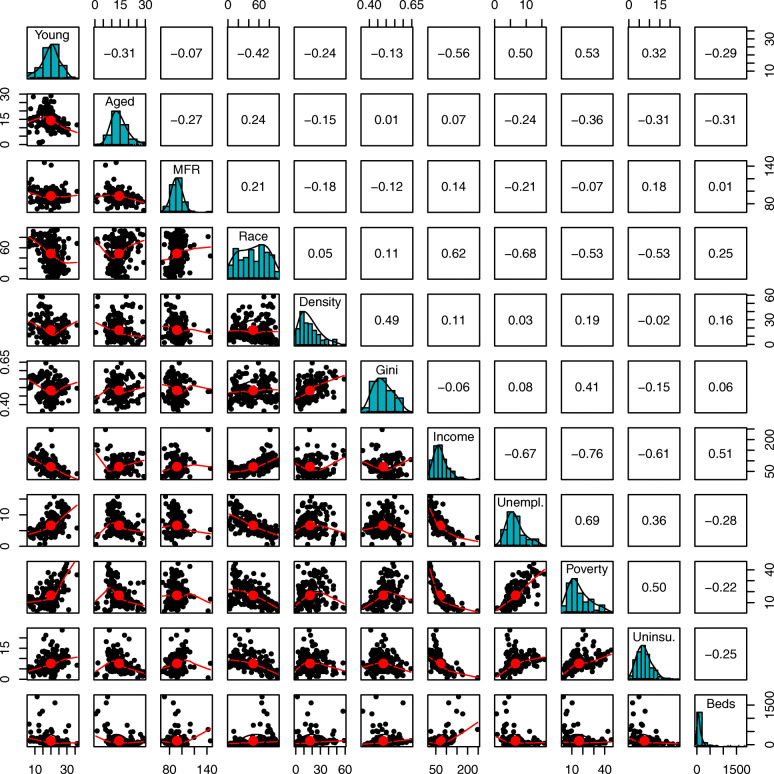
Panel plot showing bivariate relationships between predictors. *Diagonal*: Distribution of all 11 predictor variables. *Lower:* Bivariate scatter plots. *Upper:* Pearson correlations between pairs of predictors

Spread and collinearity of the predictors was assessed through histograms, bivariate scatterplots, and Pearson correlation coefficients. The strongest collinearities existed between income, poverty, and unemployment. There was only one bivariate correlation above 0.7 (median household income and poverty) and none above 0.8. It was decided to leave all predictors in the analysis and to build multiple regression models in order to consider the effects of collinearity. Figure 6 shows panel plots of the bivariate relations between the predictors.

Table 2 shows a summary of the regression estimates from the different regression models investigated. In particular, four predictors appear significant in all four models: percentage of dependent youth population, race, population density, and median household income. Percentage change in the COVID-19 positivity rate per unit change in the predictors can be found from exp(*β*).

**Table 2:** Regression estimates for association of Zip Code Tabulation Area (ZCTA) level predictors with detected COVID-19 cases in New York City as at 5 April 2020.

Predictors	Univariable analysis			Multivariable analysis (full)^†^			Multivariable analysis (sig. only)^‡^			Stepwise backwards elimination		
	Estimate	95% CI	*p* value	Estimate	95% CI	*p* value	Estimate	95% CI	*p* value	Estimate	95% CI	*p* value
Demographic parameters												
Young^1^	**0.0093**	0.0057, 0.013	**<0.00**1^∗^	**0.0064**	0.0020, 0.0108	**0.00**5^∗^	**0.0076**	0.0034, 0.0117	**0.00**1^∗^	**0.0049**	0.0012, 0.0085	**0.00**9^∗^
Aged^2^	**−0.0072**	−0.0116, −0.0027	**0.00**2^∗^	−0.0002	−0.0050, 0.0046	0.915	−0.0010	–0.0054, 0.0035	0.668			
MFR^3^	0.0004	−0.0015, 0.0024	0.626	**0.0023**	0.0005, 0.0041	**0.01**2^∗^				**0.0024**	0.0009, 0.0039	**0.00**2^∗^
Race^4^	**−0.0027**	−0.0035, −0.0020	**<0.00**1^∗^	**−0.0018**	−0.0027, −0.0009	**<0.00**1^∗^	**−0.0014**	−0.0023, −0.0004	**0.00**5^∗^	**−0.0019**	−0.0027, −0.0010	**<0.00**1^∗^
Density^5^	**0.0031**	0.0012, 0.0049	**0.00**2^∗^	**0.0031**	0.0013, 0.0049	**0.00**1^∗^	**0.0023**	0.0005, 0.0040	**0.01**3^∗^	**0.0033**	0.0016, 0.0050	**<0.00**1^∗^
Economic parameters												
Gini^6^	0.2617	−0.2447, 0.7708	0.312	−0.2903	−0.7482, 0.1739	0.215				**−0.4830**	−0.8884, −0.0699	**0.02**2^∗^
Income^7^	**−0.0027**	−0.0034, −0.0021	**<0.00**1^∗^	**−0.0024**	−0.0036, −0.0013	**<0.00**1^∗^	**−0.0025**	−0.0037, −0.0013	**<0.00**1^∗^	**−0.0020**	−0.0029, −0.0012	**<0.00**1^∗^
Unemployment^8^	**0.0146**	0.0085, 0.021	**<0.00**1^∗^	−0.0051	−0.0127, 0.0027	0.194	−0.0056	−0.0132, 0.0023	0.159	**−0.0076**	−0.0146, −0.0005	**0.03**7^∗^
Poverty^9^	**0.0064**	0.0046, 0.0082	**<0.00**1^∗^	−0.0032	−0.0072, 0.0009	0.120	**−0.0047**	−0.0084, −0.0010	**0.01**4^∗^			
Health parameters												
Uninsured^10^	**0.0154**	0.0110, 0.0200	**<0.00**1^∗^	0.0031	−0.0023, 0.0085	0.255	**0.0064**	0.0013, 0.0115	**0.01**4^∗^			
Beds^11^	−0.014	−0.0306, 0.0023	0.090	**−0.0180**	−0.0314, −0.0046	**0.00**8^∗^				**−0.0198**	−0.0326, −0.0071	**0.00**2^∗^

^1^Percentage of population under 18

^2^Percentage of population 65+

^3^Males per 100 females

^4^Percentage of population that identify as white (alone or in combination with another race)

^5^Population density

^6^Gini index

^7^Median household income in $1,000s

^8^Percentage of working age population unemployed

^9^Percentage of population living below the poverty threshold

^10^Percentage of population uninsured

^11^ log(total number of hospital beds per 1000 people within 5 km)

^*^Significant at *α*=0.05

^†^All predictors

^‡^Only significant predictors from the univariable step

Concerning youth dependency (*Young*), a 5% increase in the percentage of young population leads to an increase in COVID-19 positivity rate of 4.8% (95% CI 2.9 to 6.7%, *p*<0.001) in the univariable model, an increase of 3.3% (95% CI 1.0 to 5.5%, *p*=0.005) in the full multivariable model, an increase of 3.9% (95% CI 1.7 to 6.0%, *p*=0.001) in the partial multivariable model, and an increase of 2.5% (95% CI 0.6 to 4.3%, *p*=0.009) in the stepwise backwards elimination model. Concerning race (*Race*), a 10% decrease in the white population leads to an increase in COVID-19 positivity rate of 2.8% (95% CI 2.0 to 3.5%, *p*<0.001) in the univariable model, an increase of 1.8% (95% CI 0.9 to 2.7%, *p*<0.001) in the full multivariable model, an increase of 1.4% (95% CI 0.4 to 2.3%, *p*=0.005) in the partial multivariable model, and an increase of 1.9% (95% CI 1.0 to 2.8%, *p*<0.001) in the stepwise backwards elimination model. Concerning population density (*Density*), an increase of 10,000 people per km^2^ leads to an increase in COVID-19 positivity rate of 3.1% (95% CI 1.2 to 5.0%, *p*=0.002) in the univariable model, an increase of 3.2% (95% CI 1.3 to 5.0%, *p*=0.001) in the full multivariable model, an increase of 2.3% (95% CI 0.5 to 4.1%, *p*=0.013) in the partial multivariable model, and an increase of 3.4% (95% CI 1.6 to 5.1%, *p*<0.001) in the stepwise backwards elimination model. Finally, concerning income (*Income*), a $10,000 decrease in median household income leads to an increase in COVID-19 positivity rate of 2.8% (95% CI 2.1 to 3.4%, *p*<0.001) in the univariable model, an increase of 2.5% (95% CI 1.3 to 3.6%, *p*<0.001) in the full multivariable model, an increase of 2.6% (95% CI 1.3 to 3.8%, *p*<0.001) in the partial multivariable model, and an increase of 2.1% (95% CI 1.2 to 2.9%, *p*<0.001) in the stepwise backwards elimination model.

### Final model

**Fig. 7:**
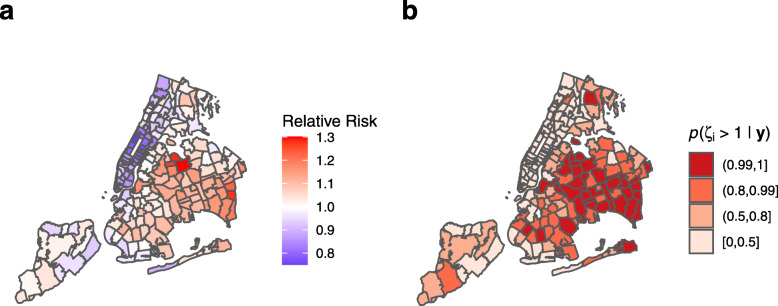
Ecological regression model for COVID-19 cases in New York City by Zip Code Tabulation Area (ZCTA). As at April 5, 2020, final Poisson BYM2 model including percentage of young population, percentage of population identifying as white (alone or in combination with another race), population density, and median household income as predictors. **a** Area-specific relative risk, *ζ*_*i*_. **b** Posterior probability for relative risk, *p*(*ζ*_*i*_>1|**y**)

**Fig. 8:**
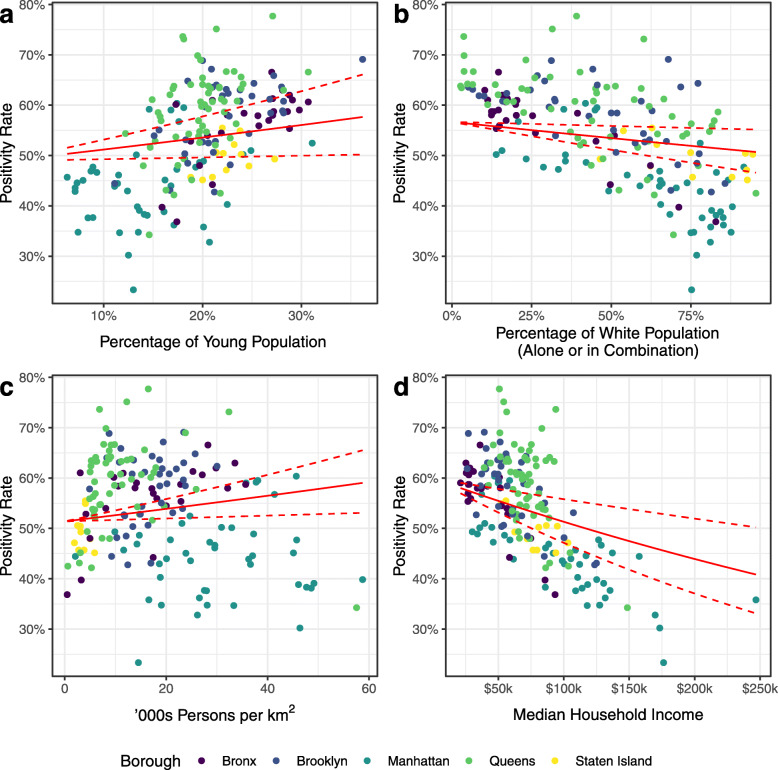
Positivity rate for total COVID-19 tests in New York City by Zip Code Tabulation Area (ZCTA) against predictors used in final model. As at 5 April 2020, using final Poisson BYM2 model. Red regression lines show model estimates and 95% confidence interval (CI) with other predictors held at their mean values. **a** Percentage of young population. **b** Percentage of population that identify as white (alone or in combination with another race). **c** Population density. **d** Median household income

A final model was built using percentage of young dependent population (*Young*), race (*Race*), population density (*Density*), and median household income (*Income*) as predictors. Table 3 shows a summary of the regression estimates from this model. Figure 7a shows the area specific relative risk *ζ*_*i*_ for this model, while Fig. 7b shows the posterior probability that the relative risk is greater than 1, *p*(*ζ*_*i*_>1|**y**). In this model, a 5% increase in the young population leads to a 2.3% (95% CI 0.4 to 4.2%, *p*=0.021) increase in COVID-19 positivity rate. A 10% decrease in the white (alone or in combination with another race) population leads to a 1.2% (95% CI 0.3 to 2.1%, *p*=0.021) increase in COVID-19 positivity rate. A 10,000 person per km^2^ increase in population density leads to a 2.4% (95% CI 0.6 to 4.2%, *p*=0.011) increase in COVID-19 positivity rate. A $10,000 decrease in median household income leads to a 1.6% (95% CI 0.7 to 2.4%, *p*<0.001) increase in positivity rate. Figure 8 shows the positivity rate for COVID-19 by ZCTA against each of these predictors, along with our regression estimates and CIs.

**Table 3:** Regression estimates for final model of association of Zip Code Tabulation Area (ZCTA) level predictors with detected COVID-19 cases in New York City as at 5 April 2020

Predictors	Estimate	95% CI	*p* value
Young^1^	**0.0045**	0.0007, 0.0083	**0.02**1^∗^
Race^2^	**−0.0012**	−0.0021, −0.0003	**0.01**0^∗^
Density^3^	**0.0024**	0.0006, 0.0041	**0.01**1^∗^
Income^4^	**−0.0016**	−0.0024, −0.0007	**<0.00**1^∗^

^1^Percentage of population under 18

^2^Percentage of population that identify as white (alone or in combination with another race)

^3^Population density in ’000s persons per km^2^

^4^Median household income in $1,000s

^*^Significant at *α*=0.05

### Race

To further investigate the significant predictor race, we conducted additional modeling efforts and divided *Race* into five racial groupings: White, Black or African American, Hispanic, Asian, and Other (including American Indian and Alaska Native, Native Hawaiian and Other Pacific Islanders, Caribbean, and Mixed Race). We ran the final model five times which each of these racial groups considered explicitly one at a time. Table 4 shows a summary of the regression estimates from these models. In all cases, the significance of the other three predictors (*Young*, *Density*, and *Income*) was unchanged.

**Table 4:** Regression estimates for models including each one of the five different race categories (one at a time). All models also included young population (*Young*), population density (*Density*), and medium household income (*Income*) as predictors, which were always significant (as they were in the final model reported in Table 3)

Race	Estimate	95% CI	*p* value
White^1^	**−0.0018**	−0.0027, −0.0008	**<0.00**1^∗^
Black^1^	**0.0011**	0.0003, 0.0018	**<0.00**1^∗^
Hispanic^1^	0.0002	−0.0008, 0.0012	0.718
Asian^1^	0.0000	−0.0013, 0.0014	0.966
Other^1^^†^	0.0015	−0.0035, 0.0064	0.588

^1^Percentage of population identifying as given race

^*^Significant at *α*=0.05

^†^Includes American Indian and Alaska Native, Native Hawaiian and Other Pacific Islanders, Caribbean, and Mixed Race

**Fig. 9:**
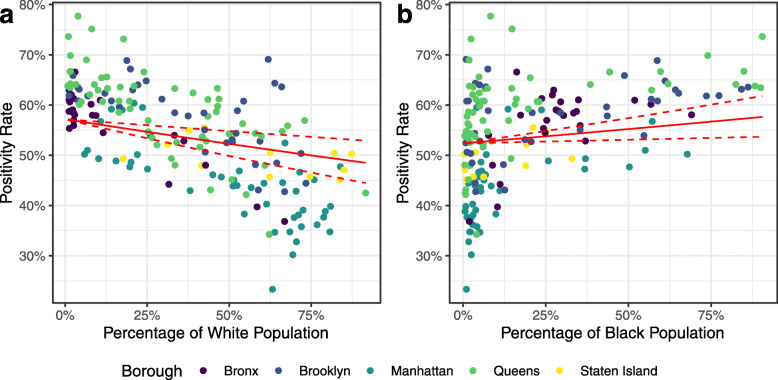
Positivity rate for total COVID-19 tests in New York City by Zip Code Tabulation Area (ZCTA) as a function of race. As at 5 April 2020, Poisson BYM2 models incorporating explicit racial groupings along with young population (*Young*), population density (*Density*), and median household income (*Income*) as predictors. Regression lines show model estimates and 95% confidence interval (CI) with other predictors held at their mean values. **a** Percentage of population identifying as white. **b** Percentage of population identifying as Black

We found race (*Race*) to be significant for proportion of White population (*p*<0.001) and Black population (*p*<0.001), but not for Hispanic (*p*=0.718), Asian (*p*=0.966), or Other (*p*=0.588) populations. A 10% decrease in the White (alone) population leads to a 1.8% (95% CI 0.8 to 2.8%) increase in the positivity rate, while a 10% increase in the Black population leads to a 1.1% (95% CI 0.3 to 1.8%) increase in the positivity rate. Figure 9 shows the positivity rate for COVID-19 by ZCTA as a function of the percentage of White and Black populations, along with our regression estimates and CIs.

## Discussion

During the opening stages of the COVID-19 pandemic in NYC, there was considerable variation in detected cases between neighborhoods in the city. Disease mapping shown in Fig. 5 displays a number of high risk areas, notably around Corona, Southeast Queens, East Bronx, and the orthodox Jewish community around Borough Park, Brooklyn. The unprecedented national response included a large number of media stories touting various covariates as predictors of either COVID-19 cases or mortality. In this ecological study, we attempted to use spatial modeling techniques to assess the association between number of COVID-19 cases detected in different neighborhoods of NYC and neighbourhood-level predictors. Our findings indicated a significant direct association between detected cases and the proportion of young dependents in the population as well as population density. We also found a significant inverse relationship between detected cases and median household income. We further found a significant positive association between COVID-19 cases and the proportion of the population identifying as black, and conversely, an inverse relationship with the proportion of the population identifying as white. We did not find a consistently significant relationship between detected cases and the other potential predictors; even those such as poverty, unemployment, and lack of insurance that were significant in a univariable model.

Our findings indicate statistically significant associations between three of the five demographic predictors included in the study. We find percentage of young dependents in the population to be a statistically significant predictor in all of the models in which it appears as a factor. Conversely, we find that the aged percentage of the population (65+) is not consistently a significant predictor of COVID-19 test positivity rate. This is congruent with evidence from Chan et al. [14] and Bai et al. [15], both of whom suggest significant transmission by young asymptomatic carriers. We further hypothesize that attitudes and behavioral patterns could play a significant role in this effect. As an example, increasing mortality of COVID-19 with age has been well publicized, and we suggest this may incline older communities to adhere to preventative public-health measures more. Conversely, the same information may be interpreted by younger populations that they are not at significant risk, potentially encouraging riskier behaviors. We found that high density population is a significant predictor of increased COVID-19 test positivity rate. These results support multiple studies of the current pandemic [37–39] that found that contact rates in well-mixed populations are proportional to population density. In the extreme scenario, the influence of high population density was seen in the rapid spread of the virus on cruise ships, notably the Diamond Princess, in late January 2020 [40, 41]. Hu et al. use kinetic theory of Van der Waals gas models to show that population contact rates increase with population density (to a saturation limit) [42]. These increased contact patterns in higher density neighborhoods, combined with disease transmission through respiratory droplets [43] likely leads to increased positivity rates.

Race (White/non-White) was a consistent significant factor in our original statistical analysis. When we examined race in greater detail, we found significant associations between COVID-19 positivity rate and the proportions of the population identifying as Black (positive association) or White (negative association), but not Hispanic, Asian, or Other. There has been much reporting on disparities in COVID-19 influence due to race [17]. The confounding sociological relationships between race and economic affluence are well established [44], with African Americans more likely to live in densely populated, low-income neighborhoods, leading to increased contact patterns [45]. Further, the higher incidence of concomitant comorbidities among African American populations (including hypertension, diabetes, obesity, and cardiovascular disease) [46] may lead to an increase in symptomatic cases. Other cohort studies have also shown differences in racial groups that we combined into our *Other* category [47]. Due to the low number of cases associated with these minority racial populations, we chose not to further divide our race groups, which could increase the risk of ecological fallacy with our aggregate methodology [48].

While the balance of males and females was not consistently significant as a factor, we found some evidence that areas with more males are associated with higher detected COVID-19 cases. Wenham et al. [49] note the lack of sex analysis by global health institutions. Studies have posited sex differences in immunological function [50] or smoking prevalence/pattern [51] as potential causes of differing medical outcomes. We found no studies to date examining sex specific behavior trends in relation to COVID-19 transmission and incidence. Looking back further, we found conflicting evidence from studies on the 2009 H1N1 pandemic. Some studies suggested that females were more willing to engage in public health precautions [52], while others suggested no significant sex effects [53]. We suggest that further studies be undertaken to consider whether sex specific behavioral, employment, or other trends are mechanisms that could explain sex effects on positivity rates.

Regarding the economic predictors, we note that our findings are in agreement with a previous, non-pandemic study [54], which found that affluence (in our case household income) was a significant predictor on self-rated health while poverty and income inequality (the Gini index) were not significant factors. Wen et al. suggest that the presence of affluence sustains neighborhood social organizations, which in turn positively affect health. If we extend this argument to the current pandemic, we could hypothesize that these social organizations further act to pass on information and promote community adoption of transmission-reduction policies such as social distancing [55]. Furthermore, we note that those in low affluence neighborhoods are more likely to live in higher density residence arrangements, for example community housing and shared family dwellings, contributing to transmission of the virus among the neighborhood [40]. While previous studies [56] have found influence of unemployment on disease transmission, we note that the unprecedented shutdown of national infrastructure and the economy has meant that many previously employed people suddenly found themselves either unemployed, furloughed, or working from home. In a short period of time, this drastic measure has completely altered the employment landscape of NYC such that it is unsurprising that the unemployment figure from 2018 is not significant.

We found that neither of our healthcare-related predictors was consistently significant. Lack of insurance has previously been a barrier to both diagnosis and treatment [57, 58]. However, in the COVID-19 pandemic, significant state resources were directed such that testing was freely available to all eligible New York residents. Furthermore, testing became freely available to all USA residents on 18 March 2020, as a result of the Families First Coronavirus Response Act (H.R. 6201) [59]. Given the unprecedented free access to testing, it is unsurprising that lack of insurance was not a significant predictor by 5 April when the data were collected. We hypothesize that conducting the same analysis on detected cases prior to 18 March could potentially draw different conclusions about the significance of insurance. Unfortunately, the data on detected cases by ZCTA only became publicly available from NYC DOHMH on 1 April and did not include temporal granularities prior to that date.

In addition to the four predictors in our final model, we also considered collinearity of the remaining predictors by conducting a principal component analysis (PCA). We generated a single social deprivation metric encompassing unemployment, poverty, and lack of insurance, all of which had a reasonable degree of correlation (we did not include race or income since they were significant on their own). We conducted similar regression approaches using this metric; however, it was only significant in the univariable case (*p*<0.001).

We note five key limitations of the ecological study. First, our dependent variable is the number of detected COVID-19 cases, which may be significantly different from the number of true cases [60]. We believe, however, that this does not detract from the validity of the study, since characterization of the detection and prevalence is important for pandemic management [61]. Studies on HIV rates among at risk populations suggest that the relationship between predictors and the number of detected cases is likely a complex interaction via at least three pathways: the true number of cases, access to testing (means) [62], and population attitudes to testing (motivation) [63, 64]. Thus, we can still develop valid inferences, even if we cannot elicit with certainty which one (or ones) of these pathways the significant predictors act through. This limitation also incorporates natural selection bias in the dependent variable, in that there is a self-selecting group of the population who choose to be tested for COVID-19 (for example due to the presence of symptoms or known contact with an infected person). This group, captured by the total COVID-19 tests, may have different characteristics to the total NYC population (one example could be young people being more likely to get tested). By using the total number of COVID-19 tests as our exposure, we limit the scope to inferences about the test positivity rate, and we further caution that this should not be used as an unbiased estimator of total COVID-19 incidence [65]. Second, any associations made must be interpreted with caution since, as with any observational study, spurious correlations produced by unstudied confounding factors may be present. Caution is also advised due to the ecological fallacy of making individual inferences from aggregate data. Further verification is required to determine true causative links between predictors and detected cases even when associations are significant. Third, the significant predictors found are likely not the only explanations for different positivity rates between different neighborhoods. However, this study does provide useful insight into explaining between-neighborhood variation. Fourth, since testing has been coordinated within the city limits at the borough level, there may be borough-level biases related to COVID-19 testing. However, if these biases exist, they likely inhibit testing access in low-income neighborhoods [66, 67] such that the inverse association found between income and positive cases is more pronounced than what the model suggests.

Finally, in our spatial model, we used an ICAR adjacency matrix of first-order lag points, i.e., a nearest neighbor structure where two ZCTAs are considered connected if (and only if) they share a border. An argument can be made that, in a highly mixed urban environment such as NYC, this structure, shown in Fig. 4d, does not adequately capture the spatial heterogeneity. However, there is sparse literature on the application of different neighborhood structures to BYM models [68, 69]; Rodrigues and Assunção argue that this is primarily due to the ease of nearest neighbor implementation using geographic information systems (GIS) [70]. To investigate the effect of neighborhood mixing, we created an additional series of lagged adjacency matrices from second- through fifth-order implying increasing levels of connectivity. We ran all our model simulations (univariable, multivariable, partial multivariable, stepwise elimination, and our final model) using each one of the five new adjacency matrices, generating 20 new sets of results and associated *p* values. In all cases (i.e., all neighborhood connectivities), the main study conclusions were unaltered; in particular, young dependent population, race, and income were still significant predictors in all models. The significance of population density however did decline with increased mixing, ceasing to be significant above third-order connectivity in our final model.

## Conclusions

Within the constraints imposed by the limitations of an ecological analysis, we conclude that there exist consistent, significant associations between COVID-19 test positivity rate and the percentage of young dependents in the population as well as population density. Further, there is also a significant association between COVID-19 test positivity rate and low income neighborhoods. Finally, there is a significant association between neighborhoods with a large percentage of black population or a low percentage of white population and COVID-19 test positivity rate. The significance of young dependents likely comes from differing contact patterns between young and old populations. We suggest further studies to be undertaken to determine any underlying causative mechanisms to these associations, paying particular attention to willingness to engage in public health behaviors and to asymptomatic carrier transmission. We finally highlight that while predictors may change with increased time and access to testing, this study provides important insights into public health behavior in the early stages of the current and future pandemics.

## Data Availability

The datasets analyzed for this study are publicly available, a repository can be found on GitHub: https://github.com/rswhittle/NYC-COVID19-socioeconomic.

## Abbreviations

ACS: American Communities Survey
BYM: Besag-York-Mollié(model)
CI: Confidence interval
COVID-19: Coronavirus disease 2019
DIC: Deviance information criterion
DOHMH: New York City Department of Health and Mental Hygiene
H1N1: Influenza A virus subtype H1N1
NYC: New York City
PUMA: Public Use Microdata Area
ZCTA: Zip Code Tabulation Area
